# Thermal Properties of Composite Polymer Electrolytes Poly(Ethylene Oxide)/Sodium Trifluoroacetate/Aluminum Oxide (PEO)_10_CF_3_COONa + *x* wt.% Al_2_O_3_

**DOI:** 10.3390/ma12091464

**Published:** 2019-05-07

**Authors:** Miguel I. Delgado Rosero, Nori M. Jurado Meneses, Ramiro Uribe Kaffure

**Affiliations:** Physics Department, University of Tolima, Ibagué, CP 730006, Colombia; nmjuradom@ut.edu.co (N.M.J.M.); rauribe@ut.edu.co (R.U.K.)

**Keywords:** polymer electrolytes, DSC, TGA, phase transitions

## Abstract

Polymeric membranes of poly(ethylene oxide) (PEO) and sodium trifluoroacetate (PEO:CF_3_COONa) combined with different concentrations of aluminum oxide (Al_2_O_3_) particles were analyzed by impedance spectroscopy, differential scanning calorimetry (DSC) and thermogravimetry (TGA). DSC results show changes in the crystalline fraction of PEO when the concentration of Al_2_O_3_ is increased. TGA analysis showed thermal stability up to 430 K showing small changes with the addition of alumina particles. The decrease in crystalline fraction for membranes with low Al_2_O_3_ concentration is associated with the increase in conductivity of (PEO)_10_CF_3_COONa + *x* wt.% Al_2_O_3_ composites.

## 1. Introduction

Solid polymer electrolytes (SPEs) are materials that have been widely investigated for their potential use in a variety of electrochemical devices such as fuel cells, batteries, electrochromic windows, supercapacitors, among others [1]. To be used in these devices, SPEs must show high ionic conductivity, good electrochemical stability, and a wide thermal stability range [2]. Improvements in these physicochemical and structural characteristics have been reported when SPEs are added with a variety of fillers [3].

For instance, SPEs formed by poly(ethylene oxide) (PEO) and alkaline metallic salts such as Na^+^, K^+^ and Li^+^, show a reduction of the crystalline phase and an increase in dissolved ions mobility. These changes lead to systems with relatively high ionic conductivity values (σ ~ 1 × 10^−5^ S cm^−1^) [4,5]. However, the need for even higher conductivity values, such as those required in various technological applications, has led to strategies to further increase the amorphous phase by also adding plasticizers and/or ceramic materials [6,7]. 

Recent reports have shown a meaningful improvement in the electrical, thermal and mechanical properties of polymeric electrolyte membranes synthesized with the addition of inert particles of aluminum oxide (Al_2_O_3_), titanium dioxide (TiO_2_) or silicon dioxide (SiO_2_), among others [8,9,10]. As a result of their large specific surface, these inorganic oxides show strong Lewis acid interactions upon the PEO matrix that create additional hopping sites and adequate pathways for ionic motion.

Particularly, in a previous study, the electrical properties of the (PEO)_10_CF_3_COONa + *x* wt.% Al_2_O_3_ composite system were analyzed and it was found that, for low alumina concentrations, ionic conductivity increased by two orders of magnitude relative to the pure polymer [11].

The improvement in thermal, electrical and mechanical properties of electrolytes based on PEO added with ceramic particles, can be explained by the decline of the kinetics of polymer crystallization, which increases the amorphous phase in localized regions and contributes to the formation of highly conductive pathways [12,13].

Relevant information related to the fraction of crystalline phase in these systems, can be obtained from the measurement of changes in melting points and the enthalpy of phase transitions [14]. In this work, we conducted a thermal analysis of (PEO)_10_CF_3_COONa + *x* wt.% Al_2_O_3_ systems, through the differential scanning calorimetry (DSC) and thermogravimetry (TGA), to determine present phases and thermal stability and to correlate these results with those of conductivity obtained by impedance spectroscopy.

## 2. Materials and Methods 

The PEO powder (molecular weight *M*_w_ = 1 × 10^6^) and CF_3_COONa from Aldrich (Darmstadt, Germany) were vacuum dried at room temperature for 24 h and then stored in a silica gel dryer. The polymer and the salt were weighted in a 10:1 (EO:Na) ratio and then separately dissolved in acetonitrile under magnetic stirring for 4 h. The two obtained solutions were combined and stirred for 4 additional hours. Then, Al_2_O_3_, from Aldrich (~150 mesh, or <104 μm, and 50 Å of size pore, Darmstadt, Germany), were added to *x* = 0.0, 3.0, 6.0, 10.0, 20.0 and 30.0% concentrations (*x* = wt. Al_2_O_3_ 100%/(wt. Al_2_O_3_ + wt. (PEO)_10_CF_3_COONa)).

The mixture was kept on a low frequency magnetic agitation to avoid decantation of Al_2_O_3_ particles and to ensure a uniform dispersion. When the mixture reached the viscous liquid properties, it was cast on a Petri dish and then stored in a dry atmosphere to let the solvent slowly evaporate. The resulting membranes show a mechanical consistency and their thickness varies from 150 to 200 μm.

Samples were analyzed by DSC (MDSC 2920 TA Instruments, New Castle, DE, USA) from 220 to 450 K, at 10 K/min heating rate; nitrogen was used as a carrier gas. Thermogravimetric analysis were performed by a 2050 TA instruments, with 10 K/min heating rate, from 303 to 660 K using nitrogen as carrier gas.

The conductivity values were obtained by the impedance spectroscopy in a frequency ranging from 50 Hz to 5 MHz, using blocking platinum electrodes. The impedance measurements were carried out by using a HIOKI 3532-50 LCR impedance analyzer (Nagano, Japan), and the dc conductivity (σ) was calculated using the relation:

$$\sigma =\frac{l}{AR},$$

where *l* is the thickness, *A* is the area and *R* is the resistance of the sample.

## 3. Results and Discussion

**Figure 1:**
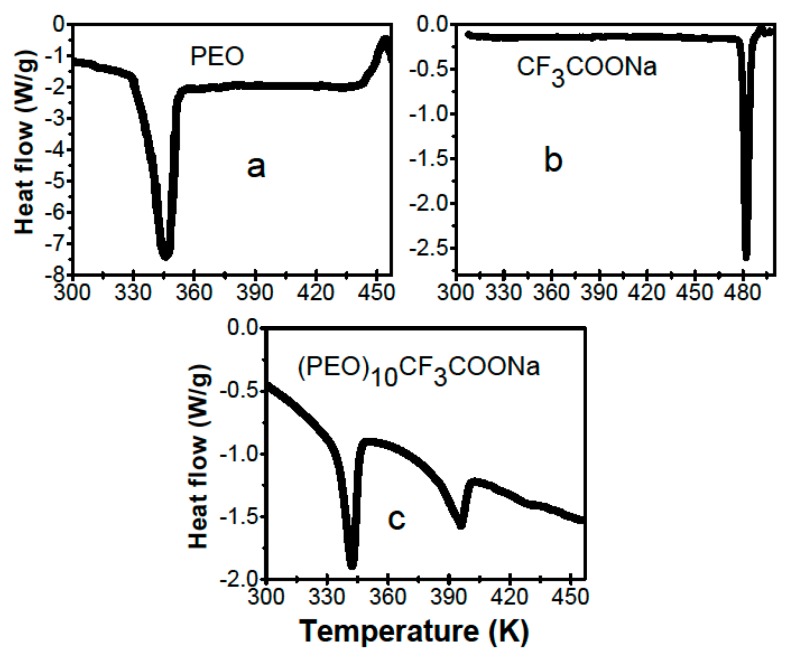
Differential scanning calorimetry (DSC) thermograph for: (**a**) pure poly(ethylene oxide) (PEO); (**b**) pure CF_3_COONa salt and (**c**) (PEO)_10_CF_3_COONa solid electrolyte.

The DSC thermogram for the pure PEO membrane is shown in Figure 1a. There, two anomalies can be observed: One endothermic about 330 K, which corresponds to the PEO crystalline phase melting, and one exothermic about 443 K corresponding to the polymer decomposition. The DSC thermogram corresponding to the CF_3_COONa salt is shown in Figure 1b; on it, an endothermic anomaly about 480 K can be observed, due to salt melting. Figure 1c shows DSC results for the solid polymer electrolyte (PEO)_10_CF_3_COONa; this thermogram shows two endothermic anomalies: One about 334 K, usual in this type of membrane and that corresponds to the PEO crystalline phase melting [15,16,17]. The other endothermic anomaly is observed about 387 K and corresponds to the melting point of a new crystalline phase of a complex formed by the combination of polymer and salt [18].

**Figure 2:**
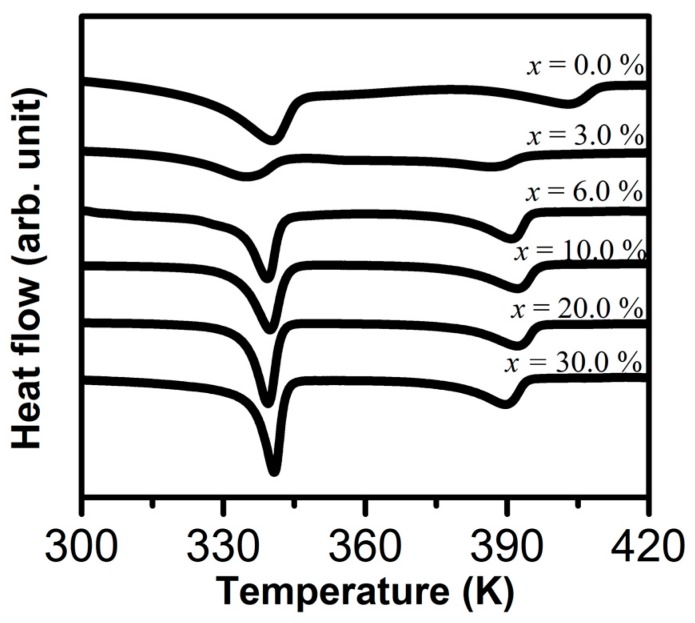
DSC thermograms for (PEO)_10_CF_3_COONa + *x* wt.% Al_2_O_3_ composite (*x* = 0.0, 3.0, 6.0, 10.0, 20.0, and 30.0).

In Figure 2, DSC thermograms of (PEO)_10_CF_3_COONa + *x* wt.% Al_2_O_3_ composite are shown for the different concentrations of Al_2_O_3_ studied. From these thermograms the values of melting temperature (*T*_m_) and enthalpy (Δ*H*_m_) are obtained; these data are given in Table 1. The relative percentage of crystallinity (*χ*_c_(%)) was calculated by:

$$\chi_{c}(\%)=\frac{\Delta H_{m}}{\Delta H_{m}^{0}}\times 100,$$

where Δ$H_{m}^{0}=$ 203 J⋅g^−1^ was used as standard enthalpy of fusion for 100% crystalline PEO [19].

For the sample corresponding to *x* = 3.0%, a significant decrease in enthalpy, and therefore in the percentage of crystallinity, is observed in relation to the sample that does not contain alumina (*x* = 0.0%). This decrease in the percentage of crystallinity in the system could be associated with the interaction between the electrolyte and Al_2_O_3_: The dispersed Al_2_O_3_ particles are coated by an amorphous material, which interrupts the alignment of the polymer chains and decreases the crystallinity of the system [20,21].

However, when the percentage of Al_2_O_3_ added is increased, i.e., for samples with alumina concentration values of *x* ≥ 6.0%, the percentage of crystallinity again increases (see Table 1). This is possibly due to Al_2_O_3_ particle aggregation that causes increases in crystallinity [22,23,24].

**Figure 3:**
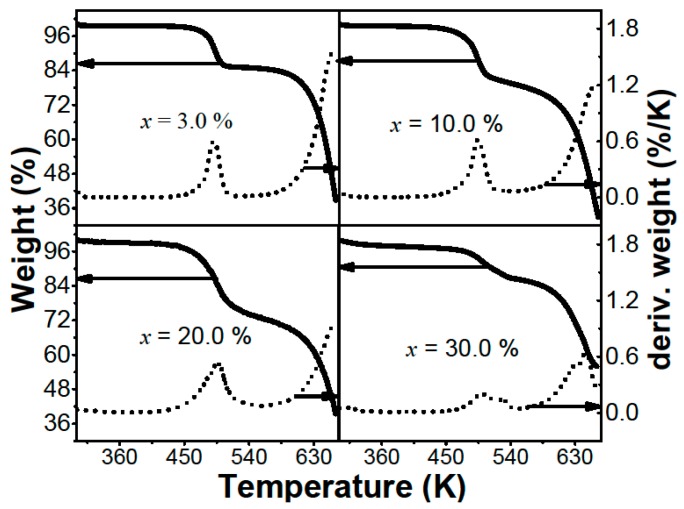
Thermogravimetry (TGA) thermographs with their derivatives (pointed lines referred to the right-hand scale) for different (PEO)_10_CF_3_COONa + *x* wt.% Al_2_O_3_ composites.

The TGA thermograms for the (PEO)_10_CF_3_COONa + *x* wt.% Al_2_O_3_ system are shown in Figure 3. Although not visible to the naked eye, in all presented thermograms (*x* = 3.0, 10.0, 20.0 and 30.0), there was a loss of mass less than 4% around 325 K. This loss corresponds to the evaporation of solvent residues (acetonitrile). Figure 3 shows the decomposition of the sample in two stages around 496 K and 663 K. Also, the maximum mass losses are shown, obtained from the derivative of mass percentage with respect to temperature (dotted line on the right-hand scale). These thermograms show that membranes are thermally stable up to 430 K, with slight variations due to the effect of the Al_2_O_3_ concentration.

**Figure 4:**
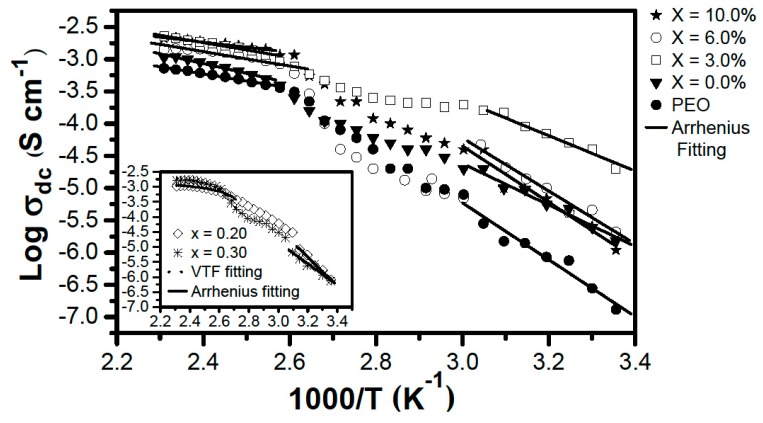
Conductivity as a function of the inverse of temperature for PEO, (PEO)_10_CF_3_COONa polymer electrolyte and (PEO)_10_CF_3_COONa + *x* wt.% Al_2_O_3_ composite. The solid line represents the Arrhenius fitting and the dotted line represents the Vogel Tammann-Fulcher (VTF) fitting.

Figure 4 shows conductivity results as a function of the inverse of temperature, obtained by the impedance spectroscopy for: Pure PEO polymer membranes, solid polymer electrolyte (PEO)_10_CF_3_COONa, and (PEO)_10_CF_3_COONa + *x* wt.% Al_2_O_3_ composites analyzed. From the graphs it can be seen that, for almost the whole temperature range analyzed, the highest conductivity values correspond to the *x* = 3.0% composite. These results are consistent with the increase of the amorphous phase around the dispersed Al_2_O_3_ particles, which would create adequate pathways to increase ionic mobility and thus improve conductivity values.

Figure 4 also shows the Arrhenius and Vogel Tammann-Fulcher (VTF) fitting in two regions: From 298 to 333 K, and from 393 to 433 K. For temperatures ranging from 333 to 393 K, it is not possible to make a fitting with either model, because the phase transitions observed in DSC occur. Parameters obtained by these fittings are in agreement with those previously reported [11].

## 4. Conclusions

DSC analyses on (PEO)_10_CF_3_COONa + *x* wt.% Al_2_O_3_ composites showed changes in the crystalline phase fraction of the system, for all concentration values of Al_2_O_3_ added. These changes in the crystallinity of the new system resulted in changes in the conductivity of the electrolyte by creating pathways to increase ionic mobility. The sample with 3.0% alumina concentration showed the highest conductivity at the same time as the highest percentage of the amorphous phase.

Thermogravimetric analyses indicate thermal stability in the membranes up to 430 K, which, together with the increase in conductivity as a function of temperature, suggests that these composites can be used as electrolyte separators in electrochemical cells such as batteries and gas sensors.

## Figures and Tables

**Table 1:** Endothermic anomaly enthalpies for different composites (PEO)_10_CF_3_COONa + *x* wt.% Al_2_O_3_.

*x* wt.% Al_2_O_3_	Melting Temperature *T*_m_ (K)	Enthalpy Δ*H*_m_ (J/g)	Crystallinity *χ_c_* (%)
0.0	319.54	67.70	33.3
3.0	320.30	26.61	13.1
6.0	332.19	53.68	26.4
10.0	332.06	56.42	27.8
20.0	332.10	58.71	28.9
30.0	335.28	62.43	30.8

## References

[1] Mindemark J., Lacey M.J., Bowden T., Brandell D. (2018). Beyond PEO—Alternative host materials for Li+-conducting solid polymer electrolytes. Prog. Polym. Sci..

[2] Judez X., Zhang H., Li C., Eshetu G.G., González-Marcos J.A., Armand M., Rodriguez-Martinez L.M. (2018). Solid Electrolytes for Safe and High Energy Density Lithium-Sulfur Batteries: Promises and Challenges. J. Electrochem. Soc..

[3] Jinisha B., Anilkumar K.M., Manoj M., Abhilash A., Pradeep V.S., Jayalekshmi S. (2018). Poly (ethylene oxide) (PEO)-based, sodium ion-conducting‚ solid polymer electrolyte films, dispersed with Al2O3 filler, for applications in sodium ion cells. Ionics.

[4] Delgado I., Castillo J., Chacón M., Vargas R.A. (2000). Ionic conductivity in the polymer electrolytes PEO/CF_3_COONa. Phys. Status Solidi B.

[5] ElBellihi A.A., Bayoumy W.A., Masoud E.M., Mousa M.A. (2012). Preparation, Characterizations and Conductivity of Composite Polymer Electrolytes. Polym. Nano Compos. Electrolytes.

[6] Liang B., Tang S., Jiang Q., Chen C., Chen X., Li S., Yan X. (2015). Preparation and characterization of PEO-PMMA polymer composite electrolytes doped with nano-Al_2_O_3_. Electrochim. Acta.

[7] Klongkan S., Pumchusak J. (2015). Effects of Nano Alumina and Plasticizers on Morphology, Ionic Conductivity, Thermal and Mechanical Properties of PEO-LiCF_3_SO_3_ Solid Polymer Electrolyte. Electrochim. Acta.

[8] Agrawal A., Satapathy A. (2015). Thermal and dielectric behaviour of polypropylene composites reinforced with ceramic fillers. J. Mater. Sci. Mater. Electron..

[9] Rybalko V.P., Nikityuk A.I., Pisarenko E.I., Kuznetsova T.I., D’yachenko P.B., Guseinov S.L., Malashin A.S., Korchmarek A.S., Kireev V.V. (2015). Effect of inorganic nanopowders on properties of acrylic composites. Russ. J. Appl. Chem..

[10] Jurado-Meneses N.M., Delgado-Rosero M.I., Meléndez-Lira M.A. (2017). Structural and vibrational studies on composites polymer electrolytes (PEO)_10_CF_3_COONa + x wt.% Al_2_O_3_. Rev. Fac. Ing. Univ. Antioquia.

[11] Jurado Meneses N.M., Delgado Rosero M.I., Vargas Zapata R.A. (2013). Conductividad iónica en nuevos compositos (PEO)10 (CF_3_COONa)-X % Al_2_O_3_. Univ. Sci..

[12] Ahn J.H., Wang G.X., Liu H.K., Dou S.X. (2003). Nanoparticle-dispersed PEO polymer electrolytes for Li batteries. J. Power Sources.

[13] Köster T.K.J., van Wüllen L. (2010). Cation-anion coordination, ion mobility and the effect of Al_2_O_3_ addition in PEO based polymer electrolytes. Solid State Ionics.

[14] Vijayalekshmi V., Khastgir D. (2018). Chitosan/partially sulfonated poly(vinylidene fluoride) blends as polymer electrolyte membranes for direct methanol fuel cell applications. Cellulose.

[15] Yang R., Zhang S., Zhang L., Bi X. (2013). Effects of LiClO_4_ on the Characteristics and Ionic Conductivity of the Solid Polymer Electrolytes Composed of PEO, LiClO_4_ and PLiAA. Mater. Sci. Forum.

[16] Gurusiddappa J., Madhuri W., Suvarna R.P., Dasan K.P. (2016). Studies on the morphology and conductivity of PEO/LiClO_4_. Mater. Today Proc..

[17] Martinez-cisneros C.S., Levenfeld B., Varez A., Sanchez J.Y. (2016). Development of sodium-conducting polymer electrolytes : comparison between fi lm-casting and fi lms obtained via green processes. Electrochim. Acta.

[18] Delgado M.I., Jurado N.M., Vargas R.A. (2012). Phase diagram of polymer electrolyte: (x)(PEO)–(1−x)CF_3_COOLi. Rev. Fac. Ing. Univ. Antioquia.

[19] Wu X.L., Xin S., Seo H.H., Kim J., Guo Y.G., Lee J.S. (2011). Enhanced Li+ conductivity in PEO–LiBOB polymer electrolytes by using succinonitrile as a plasticizer. Solid State Ionics.

[20] Polu A.R., Rhee H.W. (2015). Nanocomposite solid polymer electrolytes based on poly(ethylene oxide)/POSS-PEG (n = 13.3) hybrid nanoparticles for lithium ion batteries. J. Ind. Eng. Chem..

[21] Langer F., Bardenhagen I., Glenneberg J., Kun R. (2016). Microstructure and temperature dependent lithium ion transport of ceramic–polymer composite electrolyte for solid-state lithium ion batteries based on garnet-type Li7La3Zr2O. Solid State Ionics.

[22] Polu A.R., Kumar R. (2014). Mg2+-ion conducting poly(ethylene glycol)-TiO_2_ composite polymer electrolytes for solid-state batteries. Mater. Express.

[23] Rajesh Kumar S., Juan C.H., Liao G.M., Lin J.S., Yang C.C., Ma W.T., You J.H., Jessie Lue S. (2016). Fumed Silica Nanoparticles Incorporated in Quaternized Poly(Vinyl Alcohol) Nanocomposite Membrane for Enhanced Power Densities in Direct Alcohol Alkaline Fuel Cells. Energies.

[24] Saikia D., Chen-Yang Y.W., Chen Y.T., Li Y.K., Lin S.I. (2008). Investigation of ionic conductivity of composite gel polymer electrolyte membranes based on P(VDF-HFP), LiClO4 and silica aerogel for lithium ion battery. Desalination.

